# Left ventricular ejection fraction: clinical, pathophysiological, and technical limitations

**DOI:** 10.3389/fcvm.2024.1340708

**Published:** 2024-02-07

**Authors:** Federico Vancheri, Giovanni Longo, Michael Y. Henein

**Affiliations:** ^1^ Department of Internal Medicine, S.Elia Hospital, Caltanissetta, Italy; ^2^ Cardiovascular and Interventional Department, S.Elia Hospital, Caltanissetta, Italy; ^3^ Institute of Public Health and Clinical Medicine, Umea University, Umea, Sweden

**Keywords:** left ventricular ejection fraction, echocardiography, mitral regurgitation, aortic stenosis, aortic regurgitation, implantable cardioverter defibrillator

## Abstract

Risk stratification of cardiovascular death and treatment strategies in patients with heart failure (HF), the optimal timing for valve replacement, and the selection of patients for implantable cardioverter defibrillators are based on an echocardiographic calculation of left ventricular ejection fraction (LVEF) in most guidelines. As a marker of systolic function, LVEF has important limitations being affected by loading conditions and cavity geometry, as well as image quality, thus impacting inter- and intra-observer measurement variability. LVEF is a product of shortening of the three components of myocardial fibres: longitudinal, circumferential, and oblique. It is therefore a marker of global ejection performance based on cavity volume changes, rather than directly reflecting myocardial contractile function, hence may be normal even when myofibril's systolic function is impaired. Sub-endocardial longitudinal fibers are the most sensitive layers to ischemia, so when dysfunctional, the circumferential fibers may compensate for it and maintain the overall LVEF. Likewise, in patients with HF, LVEF is used to stratify subgroups, an approach that has prognostic implications but without a direct relationship. HF is a dynamic disease that may worsen or improve over time according to the underlying pathology. Such dynamicity impacts LVEF and its use to guide treatment. The same applies to changes in LVEF following interventional procedures. In this review, we analyze the clinical, pathophysiological, and technical limitations of LVEF across a wide range of cardiovascular pathologies.

## 1 Introduction

The assessment of left ventricular (LV) systolic function has important implications for diagnosis, follow-up, treatment, and prediction of clinical outcomes of patients with heart disease. Over the past five decades, left ventricular ejection fraction (LVEF), expressed as the stroke volume (SV) relative to end-diastolic volume (EDV), has been extensively used for quantifying LV systolic function, mostly because of the simplicity of its measurement. Clinical trials evaluating the effectiveness of different treatment strategies in HF patients also used LVEF as a threshold to categorize patients likely to benefit from therapy. It proved to have good prognostic value in HF patients, in the risk stratification of valve heart disease, and in the selection of those eligible for implantable cardioverter defibrillators (1–6). LVEF may be calculated using several cardiac imaging techniques including two-dimensional (2D) and three-dimensional echocardiography (3D), cardiac magnetic resonance (CMR), computed tomography scan (CT), and gated single-photon emission computed tomography (SPECT) myocardial perfusion imaging. However, 2D echocardiography is the most commonly used modality due to its simplicity, low cost, and widespread accessibility in clinical practices.

LVEF is widely accepted as a measure of systolic function to guide the management of several pathological conditions and to track the response to therapy. However, it has important limitations, including poor correlation with symptoms and outcomes of HF patients, being dependent on loading conditions, ventricular geometry, and image quality, that may impact inter- and intra-observer variability (7–12).

This review discusses the available evidence for clinical, pathophysiological, and technical limitations of LVEF as a measure of LV systolic function in various clinical conditions.

## 2 Clinical limitations of LVEF in HF

The limitations of LVEF as a marker of systolic LV function are particularly evident in HF patients, even in the preclinical stage. In addition to symptoms and signs, the diagnosis of HF largely relies on LVEF as a measure of cavity function, often obtained by 2D echocardiography in clinics. Most HF clinical trials have shown that the treatment benefits are mainly evident in patients with reduced LVEF (2, 13). Current guidelines recognize distinct HF patient phenotypes on the basis of LVEF: reduced LV systolic function (LVEF ≤ 40%, HFrEF), mildly reduced LV systolic function (HFmrEF), and preserved systolic function (LVEF ≥ 50%, HFpEF) (2, 14). However, there are some variations in HF classification and LVEF grading among different clinical practice guidelines (Table 1) (20–22).

**Table 1:** HF classifications according to LVEF and LVEF grading in international cardiac societies guidelines.

Society name	HF classification/LVEF grading	LVEF
ESC (2)	HF with reduced LVEF (HFrEF)	≤40%
	HF with preserved LVEF (HFpEF)	≥50%
	HF with mildly reduced LVEF (HFmrEF)	41%–49%
ACCF/AHA (15)	HF with reduced LVEF (HFrEF)	≤40%
	HF with improved LVEF (HFimpEF)	previous ≤40% and >40% at a follow-up
	HF with mildly reduced LVEF (HFpmrEF)	41%–49%
	HF with preserved LVEF (HFpEF)	≥50%
JCS/JHFS (16)	HF with reduced LVEF (HFrEF)	<40%
	HF with preserved LVEF (HFpEF)	≥ 50%
	HF with midrange LVEF (HFmrEF)	40%–<50%
	HF with recovered LVEF (HFrecEF)	LVEF improved during the treatment
	HF with worsened LVEF (HFworEF)	LVEF decreased with the treatment
	HF with unchanged LVEF (HFuncEF)	no major change in LVEF
NHFA/CSANZ (17)	HF with reduced LVEF (HFrEF)	<50%^a^
	HF with preserved LVEF (HFpEF)	≥50%
BSE (18)	Severly impaired LVEF	≤35%
	Impaired LVEF	36%–49%
	Borderline low LVEF	50%–54%
	Normal LVEF	≥55%
ASE/EACVI (19)	Male: Severely abnormal	<30%
	Moderately abnormal	30%–40%
	Mildly abnormal	41%–51%
	Normal	52%–72%
	Female: Severely abnormal	<30%
	Moderately abnormal	30%–40%
	Mildly abnormal	41%–53%
	Normal	54%–74%

^a^ If LVEF is mildly reduced (41%–49%), additional criteria are required (e.g., signs of heart failure and diastolic dysfunction with high filling pressure).

ESC, European Society of Cardiology; ACCF/AHA, American College of Cardiology Foundation/American Heart Association; JCS/JHFS, Japanese Circulation Society/Japanese Heart Failure Society; NHFA/CSANZ, National Heart Foundation of Australia and Cardiac Society of Australia and New Zealand; BSE, British Society of Echocardiography; ASE/EACVI, American Society of Echocardiography/European Association of Cardiovascular Imaging.

Based on the above classification, evidence-based therapy has proved effective in patients with HFrEF, with uncertain results in those with HFpEF being limited to certain subgroups, thus underlying the phenotypic heterogeneity of these patients (23–28). Such outcomes could be related to the fundamental differences in the pathophysiology of different groups. HFpEF patients are more often older women, likely to have cardiac and non-cardiac comorbidities, such as diabetes, hypertension, obesity, chronic obstructive pulmonary disease, and ischemic heart disease with respect to patients with HFrEF (29, 30). Patients with HFmrEF, which account for up to 20% of all HF patients, are intermediate between HFrEF and HFpEF. They include a heterogeneous population mostly with HFrEF whose LVEF partially improved with therapies and a smaller proportion with HFpEF whose LVEF declined. Patients in this range represent a transitional phenotype that may further progress either toward improvement or deterioration of LVEF (31–34).

HF, in general, is a dynamic syndrome that may progress or improve over time according to the changes in the underlying pathophysiological processes. Patients with HFrEF (LV EF <40%) may show an improvement or even normalization of LV EF, with an absolute increase of EF ≥10%, which may occur spontaneously or as a result of a good response to therapy (35). However, this change in LVEF does not necessarily mean full recovery of systolic LV function. Indeed, discontinuation of optimal therapies results in the worsening of HF (36). Over one-half of HFrEF patients, who had a myocardial infarction, as well as those with non-ischemic HErEF, demonstrate an improvement in LVEF following the resolution of myocardial stunning in the areas with residual viable myocardium or remission of the underlying pathology, such as myocarditis, peri-partum, Takotsubo, and tachycardia-related cardiomyopathies (37–39). These patients with LVEF >50% represent a different HF phenotype with recovered LVEF (HFrecEF), which from a single echocardiogram may be misdiagnosed as having HFpEF (35, 40). Evidence has shown that approximately 70% of patients with symptomatic HFpEF have recovered from low LVEF (41). Although this group has a better biomarker profile and event-free survival compared to HFrEF and HFpEF, the high rate of HF hospitalization suggests underlying function instability and high risk of HF (39) despite the fact that the normalized LVEF cannot distinguish between resolution of the underlying myocardial pathology or improvement with persistence of subclinical myocardial dysfunction. Also, despite the normalization of LVEF, in most patients, the LV systolic function remains impaired when studied by global longitudinal strain (GLS) echocardiography (42).

Accurate stratification of risk is important for the appropriate management of patients with HF (2). LVEF has been shown as a powerful predictor of fatal and non-fatal cardiovascular outcomes. Nevertheless, this is true only when the systolic function is below 45%, whereas the prognostic capability reduces significantly in patients with LVEF exceeding this threshold (43–45). Some echocardiographic models for risk prediction have been developed to identify the most significant predictors of mortality in HF patients (46, 47). While measures of LV and left atrial volume overload, as well as LV diastolic function, are independent predictors of mortality, LVEF does not significantly contribute to the risk prediction of mortality. Large studies of patients hospitalized with EF have shown similar mortality rates across the LVEF spectrum (48, 49). Also, epidemiological data show a U-shaped relationship between mortality and LVEF in patients with LVEF ≥ 65% (so-called supra-normal LV function) in whom mortality rates are similar to HFrEF (50, 51). These findings imply that categorizing systolic heart failure solely on ejection fraction may not consistently yield accurate results. GLS has been shown as a stronger predictor of cardiovascular outcomes than LVEF although it is influenced by age, sex, and loading conditions (52).

Biomarkers such as N-terminal pro-B type natriuretic peptide (NT-proBNP) have been increasingly used for prognosis and monitoring of HF therapy (53). Studies have shown that in HF patients, LVEF has no significant relationship with the prognostic value of NT-proBNP (54–56). Although overall levels of NT-proBNP are higher in patients with HFrEF compared with HFpEF, a given level of NT-proBNP is related to the same risk of death across all categories of HF, independently of LVEF. This highlights the importance of NT-proBNP in prognosis equations, irrespective of LVEF. Likewise, differences in LVEF have been shown to account very poorly for the explanation of the variation in HF-related protein biomarkers associated with inflammation, cellular proliferation, and metabolism, with a large proportion related to the underlying cause of HF, whether ischemic or non-ischemic (57–61).

### 2.1 Pathophysiological limitations of LVEF in HF

As mentioned above, LVEF is not an early marker of myocardial dysfunction and may be normal even when LV function is impaired. Several studies have shown significant impairment of myocardial function, assessed by myocardial strain and expressed as the percentage change in myocardial length measured longitudinally (GLS), circumferentially, or radially, despite preserved EF (28, 52). Such discordance is explained by changes in LV geometry which can compensate for large variations in LV function, thus maintaining overall LVEF. Accordingly, in the presence of LV dysfunction and reduced SV, LVEF may be normal in patients with LV hypertrophy and small LV cavities. In addition, LVEF does not provide pathophysiological distinctions between systolic and diastolic dysfunction. Indeed, abnormalities in diastolic function and LV filling are common in HFrEF and contribute to prognosis, while subclinical systolic impairment that can be detected by GLS is common in patients with HFpEF (62–64).

Regardless of the imaging modality used for measurement, LVEF depends on the conditions affecting SV and EDV, such as LV load conditions, myocardial contractility, and cavity dyssynchrony (7, 65). Cardiomyocytes have a limited longitudinal shortening and radial thickening during contraction of approximately 15%, which cannot account for the normal LVEF >50% (66). Experimental studies have shown that a normal LVEF is contributed to by the complex muscular structure of the LV, organized in interconnected layers with oblique spiral myofibrillar arrangement around the ventricles (67, 68). From the apex to the base, the sub-epicardial fibers are longitudinal and clockwise oriented; the sub-endocardial longitudinal and counter-clockwise fibers, and the middle layer formed by circumferential fibers which represent the thickest layer, approximately 60% of LV wall thickness (69–71). During systole, the clockwise shortening of longitudinal epicardial fibers and the counter-clockwise shortening of longitudinal sub-endocardial fibers (LV twisting) displace the LV base toward the apex, while the shortening of circumferential fibers causes LV mid-wall thickening, reducing LV cavity size (69, 72, 73). Thus, LVEF is the result of longitudinal and circumferential LV shortening.

**Figure 1:**
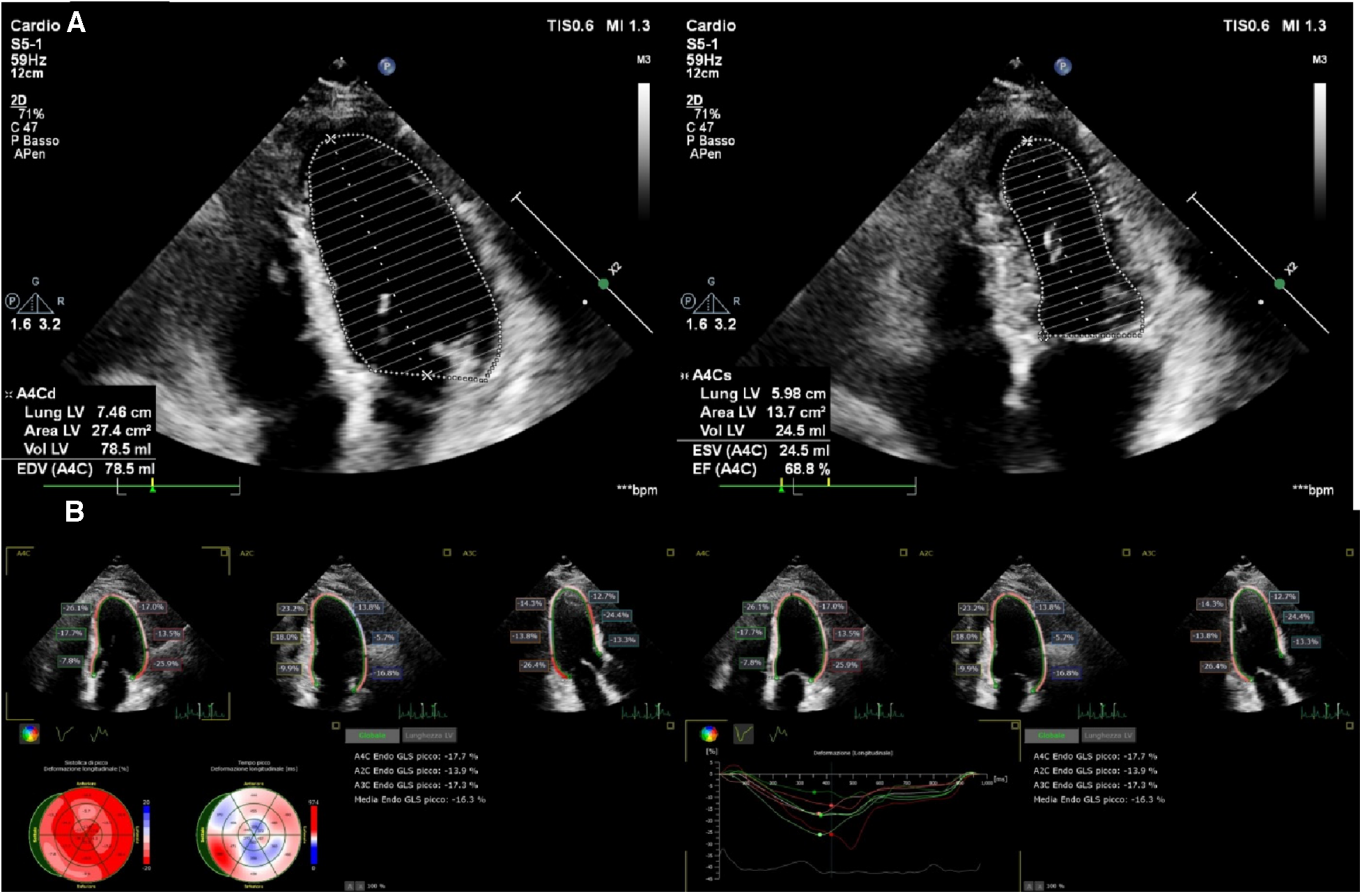
Preserved LVEF (68%) (**A**) and impaired longitudinal systolic function (GLS −16%) (**B**) in a 68-year-old man, 4 months after non-ST elevation myocardial infarction (NSTEMI).

Heart diseases have different effects on LV myocardial fiber architecture. Sub-endocardial longitudinal fibers are most susceptible to ischemia and their impairment may precede the reduction of mid-wall myocardium function, resulting in subclinical LV dysfunction that does not always affect LVEF although may be detected by GLS (Figure 1) (27, 74). Mathematical models have provided evidence that LV longitudinal shortening has a limited effect on LVEF. In contrast, the relationship between LVEF and circumferential shortening is quadratic as the short axis area is a function of the square of the radius. Hence, circumferential fibers shortening in the mid-wall has a predominant effect on LVEF with a 1 cm increase in thickness, exaggerating LVEF by approximately 13% (66, 75). Accordingly, mid-wall shortening may compensate for the impairment of longitudinal function. Moreover, the relationship of the short axis area with the square radius explains why a thick LV, as in hypertensive patients, needs less fiber shortening to produce the same LVEF compared to a thin ventricle (76–78).

## 3 Limitations of LVEF in heart valve disease

Risk stratification and timely diagnosis have critical importance in the treatment strategies of patients with valve lesions in order to improve clinical outcomes and prevent post-operative LV dysfunction. Current guidelines assign a primary role to LVEF as an independent determinant of long-term outcomes and in the early detection of LV damage in order to define the optimal timing for surgical management. However, LVEF limitation in detecting early myocardial dysfunction in asymptomatic patients has not been fully taken into account.

### 3.1 Mitral regurgitation (MR)

LVEF is influenced by loading conditions in primary and degenerative MR (intrinsic organic valve diseases, such as mitral valve prolapse or flail leaflets). Surgical valve repair or replacement is effective in relieving symptoms and preventing further progression of LV disease. However, the clinical outcome after mitral valve surgery depends largely on pre-operative LV status. Evidence exists supporting the likelihood of post-operative normalization of LV function with early valve intervention before deterioration of LV function (79, 80). Thus, optimal timing for surgery is crucial for optimum management of patients with degenerative MR.

During systole, a proportion of the LV volume is directed to the left atrium which dilates in order to accommodate the volume overload that eventually returns to the LV during the succeeding diastole. As a consequence, LV dilates and remodels, including myocardial thinning or eccentric LV hypertrophy. Volume expansion of the LV increases preload which in turn increases SV (Frank-Starling mechanism) as a result of the large end-diastolic volume, thus avoiding a relevant increase in filling pressure (81). As long as LV remodeling allows the maintenance of normal SV, LVEF remains preserved or even supernormal for a long time, erroneously suggesting a good ventricular function, although myocardial damage has already developed (82). The increase in LV volume leads to increased wall stress which is directly proportional to LV cavity radius and inversely proportional to wall thickness, according to the Laplace law. In addition, contrary to the widely accepted concept of chronic MR as a low impedance leak into the left atrium, facilitating LV emptying, the LV afterload has been shown to be normal in compensated MR and tends to increase when LV dysfunction develops (81, 83). Indeed, the regurgitant orifice is a fraction of the anatomical mitral orifice area, thus contributing to the magnitude of MR impedance.

**Figure 2:**
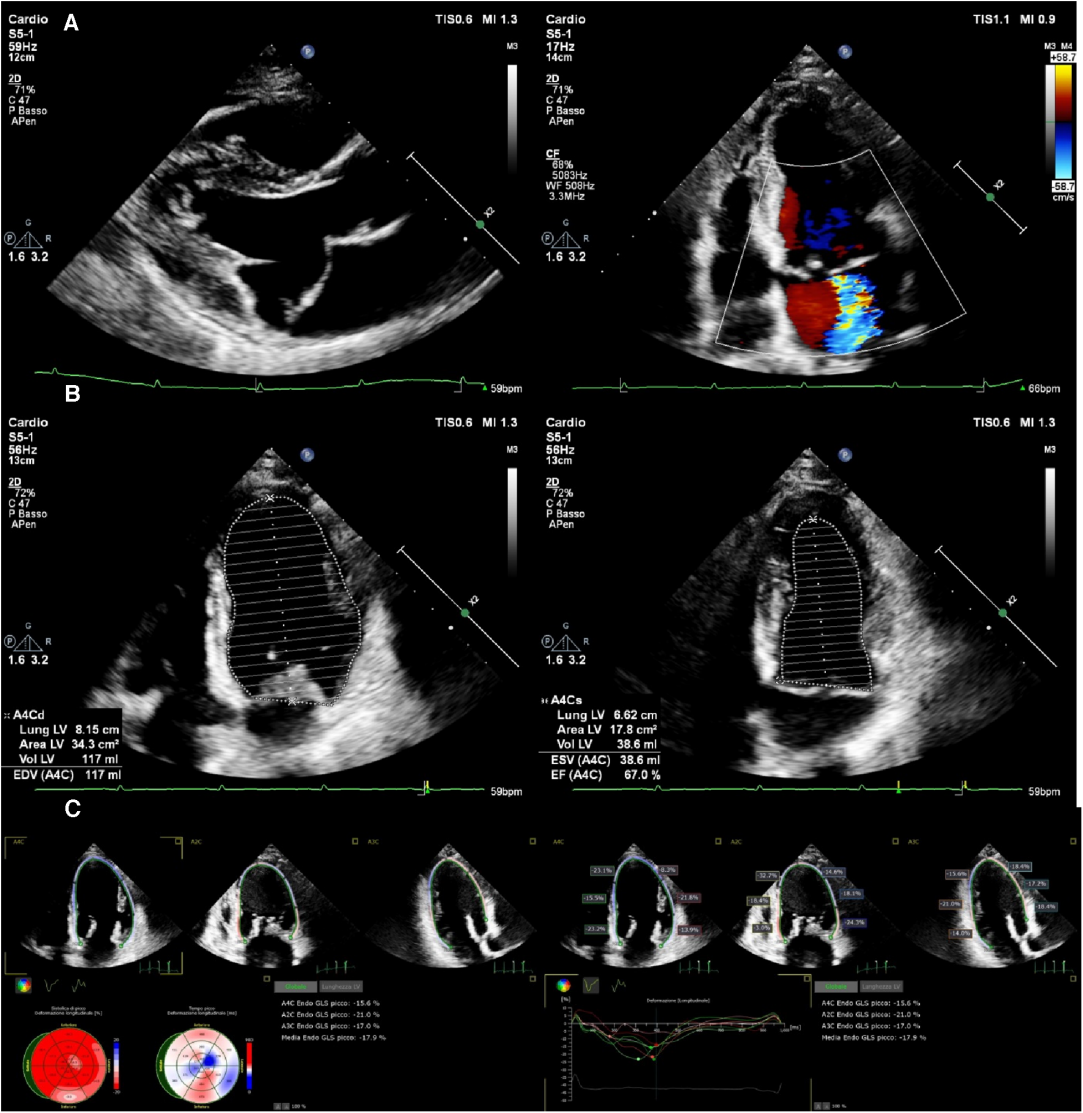
An asymptomatic 54-year-old woman with severe mitral regurgitation arising from anterior leaflet prolapse (**A**) preserved LVEF (67%) (**B**) and slightly reduced longitudinal systolic function GLS (−17.9) (**C**).

Over time, chronic volume overload and normal or increased afterload induce myofibrillar degeneration and the development of irreversible fibrosis which results in LV contractile dysfunction, as has been shown by longitudinal and circumferential strain (84–87). Even in these conditions, LVEF may be ≥60%, hiding the latent LV dysfunction, and can affect long-term survival (Figure 2) (88, 89). Indeed, it has been shown that in patients with MR and preserved global systolic function, the earliest impairment of contractility is heterogeneous and located in the LV septum, with a compensatory increase in myocardial contractile function, providing an explanation for the preserved LVEF (90).

Current guidelines recommend surgery in symptomatic MR patients. In the presence of early signs of LV systolic dysfunction, defined as LVEF ≤60% in addition to LV end-systolic diameter (LVESD) ≥ 40 mm, left atrial volume ≥60 ml/m^2^, atrial fibrillation, or systolic pulmonary arterial pressure >50 mmHg, intervention is indicated regardless of symptoms (Class I recommendation). In asymptomatic patients with preserved LV function (LVEF >60% and LVESD <40 mm), surgery is recommended if they have atrial fibrillation or pulmonary artery hypertension (Class IIa recommendation) (5, 91). In the absence of these criteria, asymptomatic patients undergo watchful waiting or active surveillance, characterized by medical management and careful follow-up of clinical and hemodynamic conditions.

The optimal management strategy for asymptomatic patients with severe MR and preserved LVEF is controversial. Because LV dysfunction based on LVEF is often underestimated in patients with severe MR, surgical decisions can be delayed. Several studies have shown that early surgical correction is associated with significant long-term survival benefits and reduced development of HF and post-operative LV dysfunction, compared to a more conservative treatment strategy (92–94). Also, although a small 5%–10% post-operative decrease in LVEF is common after mitral repair due to changes in LV volumes following withdrawal of the proportion of LV ejection contributing to regurgitation, greater LVEF reductions have been associated with irreversible myocardial dysfunction and poor prognosis. Approximately one-fifth of asymptomatic patients without Class I or IIa indications for surgery can develop severe early post-operative LV dysfunction (LVEF <50%) despite pre-operative LVEF >60% (95). Hence, watchful waiting based on LVEF follow-up may be inadequate for the early identification of impaired myocardial function due to increased fibrosis which can compromise the outcome of surgical MR repair.

### 3.2 Aortic stenosis (AS)

Calcific AS is a chronic disease characterized by a prolonged latent period where patients are relatively asymptomatic. However, when symptoms develop, they reflect LV disease and progression is usually very rapid. This is highly dependent on the response of the myocardium to the progressive reduction in the aortic valve area. The increased afterload with AS causes increased LV wall stress leading to concentric hypertrophy. This is a compensatory mechanism that results in the normalization of LV wall stress and maintenance of LVEF within the normal range (96–98). Although initially beneficial, when the hypertrophic response can no longer match the excess afterload, the LVEF begins to decline (afterload mismatch) and LV hypertrophy progresses to fibrosis. With the aging of the population, AS is increasingly becoming a syndrome that includes hypertension and arterial disease that impair LV function independently of valvar stenosis. This phenotype is characterized by hypertrophic small LV cavities, paradoxical low-flow low-gradient AS, and eventually HFpEF (99). Only surgical or transcatheter aortic valve replacement (TAVR) can prevent disease progression and poor clinical outcomes (100). Currently, expanding indications of early TAVR in asymptomatic, younger, and low-risk patients are being evaluated (101).

Current indications for AVR are based on the presence of symptoms, aortic valve gradient, and reduced LVEF (5, 91). In asymptomatic patients, AVR is recommended in LVEF <50% (Class I), with exertional symptoms (Class I), LVEF <55% (Class IIa), and LVEF >55% in addition to one of the following conditions: severe aortic stenosis (mean pressure gradient ≥60 mmHg), severe valve calcification, and markedly elevated brain natriuretic peptide (BNP).

Away from these conditions, guidelines suggest a watchful waiting strategy, delaying AVR until the onset of AS symptoms or LV systolic dysfunction (102). This strategy is based on the reported relatively low risk of sudden death in such patients, compared to the risk of surgery (103). However, a longstanding pressure overload in severe AS can cause further impairment of myocardial function, resulting in irreversible damage and development of HF, as explained above (104).

Although adaptive LV hypertrophy maintains LVEF within a normal range until end-stage disease, early subtle myocardial dysfunction may be present in AS patients (105–108). Therefore, the 50% LVEF cut-off for referral for AVR is too low because it could represent late presentation in the course of the disease. To support this view, clinical studies and meta-analyses have shown that survival in patients with AS and no or minimal symptoms at diagnosis is poor when LVEF is <60% (109–112). Moreover, in asymptomatic AS patients with LVEF >60%, early AVR has been shown to be associated with reduced all-cause mortality, acute myocardial infarction, stroke, and hospitalization for HF, compared with patients treated with conservative management and AVR deferred after symptoms onset (113–117). To avoid the latter, alternative parameters, such as GLS, may be more useful to identify early myocardial function changes and the optimal timing for AVR (118, 119).

### 3.3 Aortic regurgitation (AR)

Chronic AR causes LV volume overload and significant LV afterload (systolic wall stress) due to high stroke volume (120, 121). LV systolic function is maintained through chamber enlargement followed by eccentric hypertrophy. This pathophysiology allows for maintaining normal end-diastolic wall stress, hence normal filling pressures. In general, patients usually tolerate AR and remain compensated and asymptomatic for a long time.

Eventually, the progressive LV dilatation and the increase in LV filling pressure lead to increased systolic wall stress. This afterload mismatch and limited preload reserve of an enlarged ventricle impair cavity performance further, causing symptoms of HF with subsequently impacted survival in the absence of surgical correction (122–124). The development of symptoms also indicates irreversible LV damage caused by myocardial fibrosis, which may impede recovery of LV function after AVR (125–129). Thus, accurate detection of subclinical LV dysfunction before the development of symptoms and a reduction in LVEF should identify patients who need early surgical intervention.

Accordingly, guidelines recommend surgery, even in the absence of symptoms, for patients with a significantly dilated LV, which reflects the severity of volume overload (LVESD >50 mm or >2.0 mm/m^2^) even in the presence of preserved LVEF which overestimates the real EF (5, 91). Also, studies have shown that more than 80% of deaths in asymptomatic patients occur before reaching the guideline-recommended threshold for surgical intervention (130, 131). Hence, as in other settings of subclinical dysfunction, LVEF is not a sensitive marker of early LV systolic dysfunction compared with longitudinal and circumferential LV strains in identifying patients with impaired LV function, who require AVR, despite preserved LVEF (132–134).

## 4 Limitations of LVEF in the selection of patients for implantable cardioverter defibrillator (ICD)

Patients with recent myocardial infarction (MI) and LV dysfunction, as well as those with non-ischemic LV systolic dysfunction, are at high risk of sudden cardiac death (SCD), frequently due to ventricular arrhythmias (135, 136). ICD implantation has proved to be effective in reducing the occurrence of SCD in these patients (137–141). The current risk stratification strategy for SCD primary prevention is largely based on the measurement of LVEF function. An LVEF ≤35%, despite optimal medical therapy, is the currently recommended threshold for Class I ICD implantation for the primary prevention of SCD and all-cause mortality in symptomatic patients with HF of ischemic origin, and Class IIa for patients with HF of non-ischemic origin (2). The strongest evidence for benefits from ICD implantation according to LVEF <35% has been observed only in patients with ischemic HF, which resulted in long-term reduction of all-cause mortality (141).

Although LVEF is the most powerful non-invasive assessor, compared to electrocardiographic techniques, for identifying HF patients who are at risk of SCD, it has limited sensitivity (142). Population-based studies have shown that the majority of SCD cases occur in individuals who had no known heart disease or had heart disease with normal or only moderately impaired LVEF function (143–146).

In addition, over the last two decades, there has been a decline in the risk of SCD, especially in patients with non-ischemic HF, attributable to an improvement in medical therapy (147–150). Clinical trials using sacubitril-valsartan (ARNI) and sodium-glucose co-transporter 2 inhibitors (SGLT2) have demonstrated a consistent reduction in ventricular arrhythmias and SCD due to their anti-arrhythmic properties in addition to their ability to improve LVEF, thus reducing the eligibility for ICD implantation based on LVEF ≤35% (151–154).

Patients with ischemic HF are at high risk of mortality early after MI. However, early measurement of LVEF does not take into account the spontaneous improvement of LVEF following the recovery of myocardial stunning occurring in more than half of post-MI patients (37). Hence, a month after MI, there may be no longer an indication for ICD implantation because of the rise in LVEF >35%. However, approximately two-thirds of SCD occurs in patients with LVEF >35% (155, 156).

Thus, LVEF alone is inadequate as a marker of the underlying myocardial damage predisposing to SCD because it has no causal relationship with the mechanisms of arrhythmia (146). LV dysfunction and remodeling both in ischemic and non-ischemic HF result in myocardial fibrosis (157, 158). Even in the absence of contractile impairment, fibrosis induces electrophysiological heterogeneity which promotes the development of ventricular arrhythmias (159, 160).

More accurate strategies to identify patients at risk include the quantification of myocardial fibrosis using mechanical dispersion at strain imaging and cardiac magnetic resonance imaging with late gadolinium enhancement (CMR-LGE) (157, 161–164).

## 5 Technical limitations

Most therapeutic decisions in current guidelines are based on 2-dimensional (2D) echocardiographic measurement of LVEF which uses the method of disk summation (modified Simpson's rule) to calculate LV volumes in two imaging planes (19). As an index derived from volumes, LVEF is based on the geometric assumption of an ellipsoid ventricular shape to estimate a three-dimensional volume from a two-dimensional image. This may reduce the measurement reliability when the ventricle has a non-geometric shape as occurs in patients with ischemic heart disease (165).

Poor image quality due to body shape and limited acoustic window may impair the delineation of the endocardial border and tracing of the LV cavity, leading to a foreshortened ventricle. Since echocardiography is operator-dependent, measurement inconsistencies result in large intra- and inter-observer variability, particularly in patients with reduced LVEF, which can be clinically relevant in the follow-up of LV function (4, 12). As a general rule, up to 10% variation between examinations in the same patient cannot be considered a change in LV function (9, 166, 167). Moreover, 2D measurement of LVEF shows beat-to-beat variability up to 6% and can vary with blood pressure, heart rate, and inotropic state (168).

The geometric problems of 2D echocardiography may be overcome by three-dimensional (3D) echocardiography which is less influenced by irregular geometry. However, artifacts and reduced spatial and axial resolution may limit its accuracy in estimating LVEF (169, 170). Contrast-enhanced 2D and 3D echocardiography allow accurate measurement of LV dimensions and volumes in patients with suboptimal imaging (171). CMR provides heart imaging in multiple planes with excellent endocardial definition, making it the reference standard for LVEF among non-invasive techniques. However, it is expensive, time-consuming, and not widely available in clinical practices. CT scan and SPECT are also accurate in LVEF measurement but have the disadvantage of needing contrast and radiation exposure (8).

## 6 Conclusions

Although LVEF is a widely accepted method for assessing LV function in most guidelines, it is an artificial measure with no comparable physiological measurement. It only describes the volumetric alterations of LV and the relationship between LV filling and ejection, which can be unrelated to myocardial systolic function. Echocardiographic assessment of LVEF plays a key role in the diagnostic and prognostic evaluation of HF patients. However, because of its sensitivity to loading conditions, EF reflects cavity systolic performance as a whole, rather than myocardial contractile function. The impact of such limitation significantly affects the right time for decision-making for valve replacement and ICD implantation, particularly in asymptomatic patients. The need for early detection of subclinical changes in systolic function and myocardial performance implies the use of other parameters beyond LVEF including echocardiographic GLS and CMR. However, their routine and inclusion in guidelines require prospective randomized studies.
